# STR Markers in clinics: a rapid prenatal diagnosis by quantitative fluorescent-pcr for aneuploidies

**DOI:** 10.1186/1755-8166-7-S1-I58

**Published:** 2014-01-21

**Authors:** Sarita Agarwal, M Srinivasan, Shubha Phadke

**Affiliations:** 1 Department of Medical Genetics, SGPGIMS, Lucknow, India

## 

In the past few decades, prenatal diagnosis of fetal chromosomal abnormalities has relied on conventional cytogenetic analysis of cultured amniocytes, chorionic villi, or fetal blood. In recent years, the clinical validity of a newer technique, QF-PCR, to detect the common aneuploidies has been reported by a number of investigators. This technique has the advantage of providing rapid results for the diagnosis or exclusion of aneuploidy in chromosomes 13, 18, 21, X or Y. It is now possible to choose standard chromosome analysis or QF-PCR for the prenatal diagnosis of chromosomal abnormalities, or to perform both tests, depending on the clinical indication for testing.

QF-PCR exploits the distribution of STR markers, 2-6bp tandem repeats, across the genome. This DNA marker is easily amplified by polymerase chain reaction (PCR) without the problem of differential amplification; that is, the PCR products for STRs are generally similar in amount, making analysis easier. An individual inherits one copy of an STR from each parent, which may or may not have similar repeat sizes. The number of repeats in STR markers can be highly variable among individuals, which make these STRs effective for diagnosis and human identification purposes. It has an added advantage of identifying maternal or paternal origin of nondysjunction.

The establishment of QF-PCR has been initiated with the aim of providing rapid prenatal diagnostic service and to reduce anxiety of patients about their at risk pregnancies.

For validation, we performed analysis on 100 confirmed cases of Down syndrome with the markers D21S1435, D21S11 and D21S1411, whose heterozygosity has been studied in North Indian population, with heterozygosity of 70.1%, 83.1% and 93.6% respectively. We observed 100% concordance with the clinical diagnosis as well as cytogenetic analysis. With these results we extended the methodology in prenatal services were chromosomal studies are very common for suspected Down syndrome pregnancies. However, in addition we included 2 more markers D21S1411 and D21S1413 for 21 chromosome, D13S238 and D13S631 for 13 chromosome, D18S391 for 18 chromosome and AMEL, XHPRT, X22 and SRY for sex chromosomes. Addition of 13, 18 and sex chromosomes marker were helpful at drawing conclusive reports for maternal contaminated samples and simultaneously to screen for rare aneuploides like 13, 18 trisomy and Klinefelter syndrome.

The test was offered to the pregnancies with triple test positive as well as abnormal findings in ultrasonography. The source of DNA was 3 – 5 ml of amniotic fluid while in few cases chorionic villi sample. 190 pregnancies were studied to date, 3 pregnancies (1.6%) were turned to be Down syndrome positive while rest was negative for Down syndrome while 1 case was turned out to be homozygous for all 4 markers of 21chromosome in which we required additional markers to do reporting. However, karyotying showed this sample to be negative for Down syndrome. Rest all the finding was consistent with that of karyotyping results.

The technique has its own advantage that can utilize the maternal blood contaminated samples, to add more, low volume of sample requirement and possibility to do reporting within 6 – 12 hours. Still we are working to record heterozygosity of rest of the 8 markers and to tune up the marker panel accordingly.

